# A Case of Sarcoidosis Mimicking Lymphoma Confounded by Cognitive Decline

**DOI:** 10.7759/cureus.13667

**Published:** 2021-03-03

**Authors:** Benjamin S Bryant, Kathleen A Marsh, William J Beuerlein, Darryl Kalil, Kinchit K Shah

**Affiliations:** 1 Internal Medicine, Northwestern Memorial Hospital, Chicago, USA; 2 Internal Medicine, Wake Forest Baptist Medical Center, Winston-Salem, USA; 3 Pathology, Wake Forest Baptist Medical Center, Winston-Salem, USA; 4 Internal Medicine, University of North Carolina, Chapel Hill, USA

**Keywords:** neurologic sarcoidosis, sarcoidosis-lymphoma syndrome

## Abstract

Sarcoidosis is a disease with an unknown cause that affects multiple organ systems and has a varied clinical presentation. Often, its symptomatology mimics other disease processes, such as lymphoma, tuberculosis, and amyloidosis. The reticuloendothelial involvement and typical B symptoms of weight loss, fatigue, night sweats, and lymphadenopathy can make sarcoidosis often easily confused with lymphoma. Sarcoidosis has a myriad of central nervous system (CNS) effects, which are often not recognized as symptoms of the disease. These neuropsychiatric symptoms can include, but are not limited to, cognitive decline, headaches, and personality changes. In this report, we discuss a case of a patient who presented with symptoms consistent with indolent lymphoma but was eventually diagnosed with sarcoidosis with extrapulmonary manifestations.

## Introduction

Sarcoidosis is a systemic disease process that has a varied clinical presentation. The primary manifestation of the disease is pulmonary, with 95% of patients presenting with evidence of pulmonary sarcoidosis. However, many organ systems can be involved, including the eyes, skin, lymphatics, liver, kidneys, central nervous system (CNS), and spleen. More than 40% of patients have extrapulmonary manifestations at the time of presentation. CNS involvement occurs in about 5% of patients [1]. Because sarcoidosis can simultaneously affect separate organ systems, it is known anecdotally as the “great imitator.” The myriad symptoms associated with the disease can mimic many inflammatory, infectious, and neoplastic conditions. In particular, when the reticuloendothelial system is involved, patients can experience typical B symptoms such as weight loss, fatigue, lymphadenopathy, and night sweats, which raise concern for lymphoma. Additionally, about 20% of patients with central nervous involvement and symptoms concomitantly experience psychiatric manifestations [2].

While the underlying cause of sarcoidosis is not well understood, the immune system plays a central role in its pathogenesis. Noncaseating granulomas are seen histologically, which are also associated with other pathologies such as malignancy, infection (mycobacteria, fungi, parasites, histoplasmosis), and occupational and environmental exposures (such as beryllium or silica) [3]. It is important to first rule out these disease processes before making the diagnosis of sarcoidosis.

In this case report, we present a case of sarcoidosis with initial findings suggestive of indolent lymphoma. Further history gathering and workup resulted in a diagnosis of sarcoidosis with extrapulmonary manifestations. The clinical picture was further complicated by unexplained recent cognitive decline, prompting a concomitant neurologic workup. The cognitive dysfunction was ultimately felt to be associated with sarcoidosis, with consideration given to neurosarcoidosis (NS) versus sequelae of the sarcoidosis “penumbra.” In addition to highlighting the challenges in distinguishing sarcoidosis from an indolent lymphoma, we also focus on the importance of under-recognized neuropsychiatric effects of sarcoidosis.

## Case presentation

A 65-year-old African American male with a history of chronic obstructive pulmonary disease (COPD), anemia, and heart failure with preserved ejection fraction (HFpEF) presented to the emergency department with complaints of unintentional weight loss, fevers, chills, and memory decline. He had lost 60 pounds within the past year, with associated functional decline and generalized fatigue. Within the past two months, he had developed fevers and night sweats, and within the past week, he had begun to experience cognitive decline described as being in a “fog,” which had led him to miss familiar exits on the highway and forget his way to his doctor’s office.

Upon presentation to the emergency department, his vitals included a blood pressure of 117/51 mmHg, heart rate of 95 beats per minute, respiratory rate of 18 breaths per minute, O_2_ saturation of 97%, and temperature of 98.3 °F. Physical exam showed a thin-appearing man in moderate distress secondary to shortness of breath and cognitive issues. Pertinent positives included bilateral shoddy cervical lymphadenopathy, increased respiratory effort with bilateral wheezing and inspiratory crackles, 1+ pretibial edema, and large bilateral axillary lymphadenopathy. A mini-mental status exam (MMSE) revealed a score of 24/30. Laboratory workup revealed WBC of 10.1 x 1000/mm^3^, Hemoglobin (Hb) of 9.5 g/dL, and platelet count of 400 x 1000/mm^3^. The basic metabolic panel (BMP) showed sodium of 130 mmol/L, potassium of 3.3 mEq/L, and creatinine of 0.84 mg/dL. Brain natriuretic peptide (BNP) was 225 pg/mL (normal range: 5-100). A chest X-ray showed cardiomegaly and mild interstitial edema with bilateral pleural effusions. CT brain revealed no acute findings. Given the patient’s functional decline, diffuse lymphadenopathy, and worsening mental status, he was admitted for further workup and management.

On day two of his admission, collateral information was obtained from the patient’s family, which revealed additional prior workup for his symptoms. Six months beforehand, the patient had been evaluated for worsening blurred vision and had been diagnosed with bilateral panuveitis. An extensive laboratory workup was performed, which is summarized below in Table 1.

**Table 1:** Ophthalmology uveitis panel IgG: immunoglobulin G; c-ANCA: cytoplasmic anti-neutrophil cytoplasmic antibody; p-ANCA: perinuclear anti-neutrophil cytoplasmic antibody; HEp-2: human epithelial type 2; ANA: antinuclear antibody; HIV: human immunodeficiency virus; IL-2: interleukin-2; RPR: rapid plasma reagin; HLA: human leukocyte antigen

Laboratory test	Result	Normal range
Hepatitis B surface antibody	Reactive (equivalent to >=10 mIU/mL)	
Hepatitis B core antibody	Non-reactive	
Hepatitis C antibody	Non-reactive	
Hepatitis A (IgG and IgM)	Non-reactive	
Anti-DNase B titer	96	0-300
c-ANCA	Negative	
p-ANCA	Negative	
Antinuclear Ab, HEp-2	Positive 1:160	
ANA pattern	Speckled	
Neuromyelitis optica (NMO)/aquaporin-4-IgG (AQP4) fluorescence-activated cell sorting (FACS)	Negative	
HIV antigen-antibody	Non-reactive for HIV 1 and 2	
IL-2 receptor (CD25)	2,619	<=1,033 pg/ml
Treponema pallidum particle agglutination assay (TPPA)	Non-reactive	
QuantiFERON-TB Gold Plus	Indeterminate	
Angiotensin-converting enzyme	47	8.0-53.0
cyclic citrullinated peptide antibodies	Negative	
Lyme disease serology	Negative	
Lysozyme Serum	3.35	0-2.75
RA screen	Negative	
RPR	Non-reactive	
Toxoplasma IgG and IgM	Negative	
HLA-A29	Negative	
HLA-B27	Negative	
HLA-B51	Negative	
HLA-B44	Positive	
HLA-B56	Positive	

**Figure 1:**
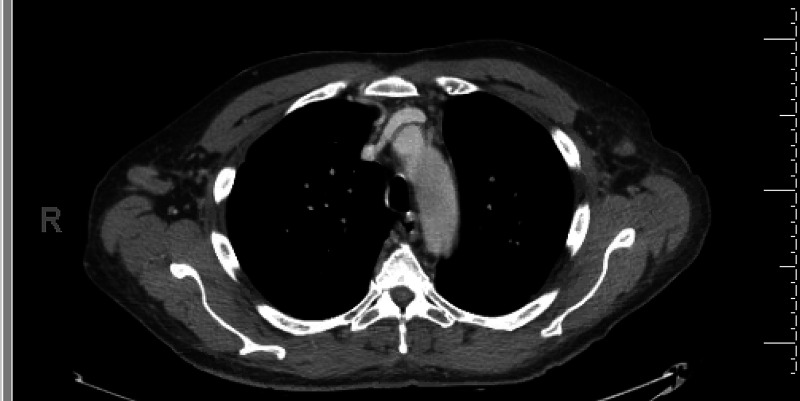
Bilateral axillary lymphadenopathy (which could be of reactive or neoplastic etiology)

**Figure 2:**
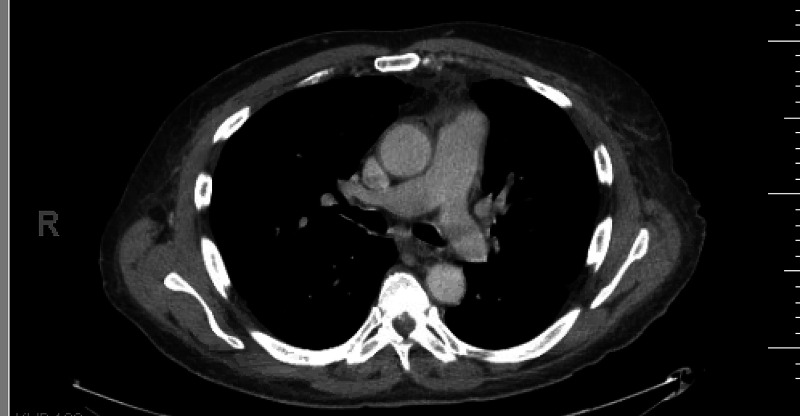
Mediastinal lymphadenopathy (which could be of reactive or neoplastic etiology)

Extensive radiology and pathology workup was performed and raised concerns for sarcoidosis. Although the patient had been prescribed methotrexate (MTX), he reported never starting it. One month prior to presentation, the patient had undergone a CT of the chest, abdomen, and pelvis with contrast, which had revealed multiple enlarged mediastinal and axillary lymph nodes and extensive bronchiectasis of bilateral lungs, with the lymphadenopathy (LAD) considered to be reactive versus neoplastic (Figure 1, Figure 2).

**Figure 3:**
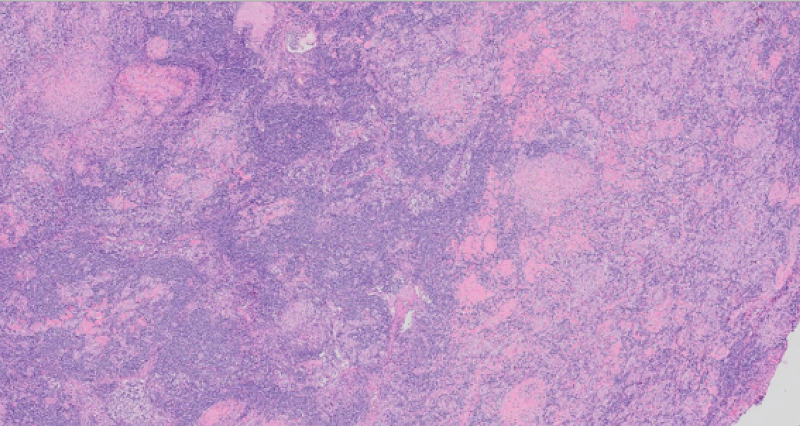
Low power demonstrates the lymph node architecture appears interrupted with rounded eosinophilic groups

**Figure 4:**
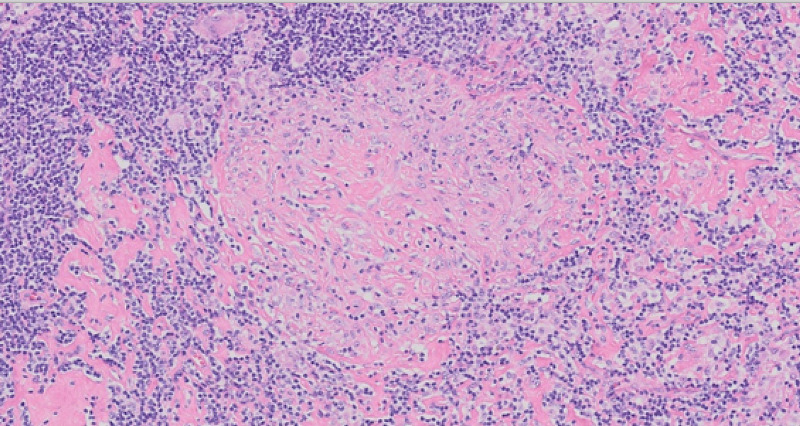
Higher power reveals that the granulomas are composed of activated epithelioid histiocytes without necrosis and surrounded by a rim of lymphocytes at the periphery

**Figure 5:**
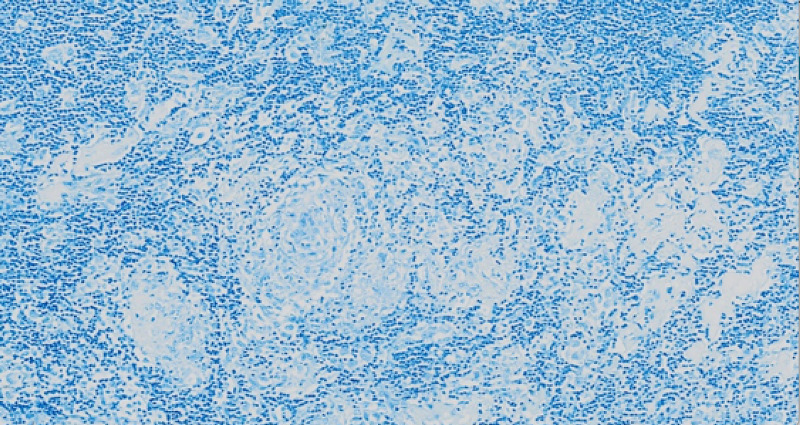
The special stain for acid-fast bacilli is negative

**Figure 6:**
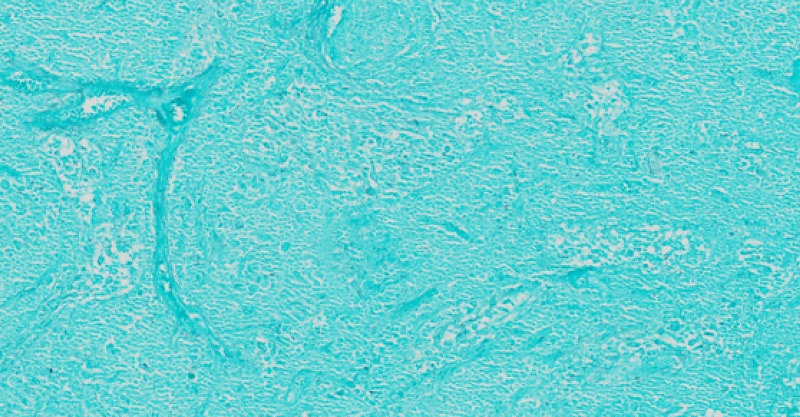
The Gomori methenamine silver (GMS) special stain is negative for additional microorganisms

Given this new information, rheumatology was consulted, inflammatory markers were drawn which showed an elevated C-reactive protein (CRP) of 50.4 mg/L (normal level: <5), and an erythrocyte sedimentation rate (ESR) of 97 mm/hr (normal range: 0-20); an excisional lymph node biopsy was pursued. Pathology from the axillary lymph node biopsy (Figure 3, Figure 4) revealed increased epithelioid histiocytes and well-formed granulomas associated with multinucleated giant cells, most consistent with sarcoidosis. We also ruled out infectious etiologies with this biopsy with acid-fast bacilli (AFB) and Gomori methenamine silver (GMS) stains, both of which were negative, as shown in Figure 5 and Figure 6. Given these results, initiating 40-mg prednisone and 15-mg MTX weekly was recommended. It is important to note that on every night during the patient’s hospitalization, he recorded a fever in the range of 101.4-102.4 °F. These fevers abated with acetaminophen and were never present during the day. Blood cultures were drawn at each instance, but they all returned free of organisms. The fevers were ultimately attributed to sarcoidosis.

Regarding the patient’s encephalopathy, neurology was consulted and an MRI brain was performed, which showed no evidence of infection, NS, CNS lymphoma, or neurodegenerative disease process. The patient’s mental status slowly improved over the course of the hospitalization, and a lumbar puncture was not pursued. In the outpatient setting, the patient was followed up with rheumatology and the steroid dose was slowly tapered. The patient also followed up with his primary care physician, stating improvements in his memory and his functional status. He has continued to live alone, retaining the ability to perform activities of daily living on his own.

## Discussion

Sarcoidosis is a systemic disease with a diverse array of clinical manifestations that can mimic many inflammatory, infectious, and neoplastic conditions. In our patient’s case, the extensive lymphadenopathy in the setting of prolonged B symptoms raised concern for indolent lymphoma. Although there is no precise definition for the diagnosis of sarcoidosis, an international consensus statement has identified three criteria [4]:

1. Compatible clinical and radiologic presentation

2. Pathologic evidence of noncaseating granulomas

3. Exclusion of other diseases with similar findings

Other laboratory values such as an elevated angiotensin-converting enzyme, hypercalcemia, and an elevated lysozyme have previously been associated with sarcoidosis, but are insufficiently specific and sensitive [5-7]. More recently, serum IL-2R has been shown to be a useful marker in diagnosing sarcoidosis with uveitis but has yet to be incorporated into the diagnostic criteria. Histologic findings of noncaseating granulomas remain the preferred modality of diagnosis.

The difficulty in distinguishing sarcoidosis from a lymphoproliferative disorder is apparent in the literature. In particular, the Bulletin of the Hospital for Joint Disease (2013) reported two similar cases of one-year histories of fatigue, fever, shortness of breath, and night sweats whose initial biopsies were consistent with sarcoidosis, but were ultimately found to have Hodgkin’s lymphoma [8]. A second case report from Hindawi included a patient who presented with symptoms of lymphoma (severe fatigue, unintentional weight loss over 18 months, and diffuse lymphadenopathy) whose lymph node biopsy was more consistent with sarcoidosis. Brincker in 1986 identified a potential coexistence between sarcoidosis and lymphoma, known as the “sarcoidosis-lymphoma syndrome,” reporting that patients already diagnosed with sarcoidosis have an increased risk (at least 5.5x greater than matched controls) of developing lymphoma [9].

In this case, the patient’s concurrent cognitive decline complicated his workup and management. Initially, the differential diagnosis for his cognitive decline included NS, meningoencephalitis, toxic-metabolic encephalopathy, tuberculous meningitis, CNS fungal infection, and dementia (Alzheimer’s versus vascular). Prior laboratory workup ruled out other causes such as neurosyphilis, HIV, herpes simplex virus (HSV), toxoplasmosis, and anti-neutrophil cytoplasmic antibody (ANCA)-related vasculitis. Given the negative MRI, clinical improvement, and biopsy-proven sarcoidosis, the differential diagnosis was narrowed, with particular focus on NS without signs of active inflammation versus parasarcoidosis, an under-reported entity. Improvement of mental status over the course of hospitalization was likely multifactorial after treatment with prednisone and MTX for sarcoidosis, fever reduction with acetaminophen, and improved nutrition.

Neurologic complaints are reported in 5-15% of all patients with sarcoidosis [10]. The diagnostic guidelines for NS were revisited in 2018 in Neurology JAMA, and three separate tiers were proposed: possible, probable, and definite NS [11].

Our patient partially met the criteria for a “probable” diagnosis of NS, given there was evidence of extra-neural sarcoidosis but with negative MRI findings. Although MRI is considered to be the most sensitive (82-97%) imaging study for NS, a study in the Quarterly Journal of Medicine highlights its imperfect nature, in that six of 54 NS cases (11%) had normal MRI findings [12]. Cerebrospinal fluid (CSF) studies were not recommended for our patient, as his mental status improved. The JAMA guidelines assert that CSF can be entirely normal in up to 30% of NS cases, and should mainly be pursued to rule out infectious or neoplastic processes.

An alternative explanation considered for our patient’s cognitive decline was parasarcoidosis, or the “sarcoidosis penumbra.” The most prevalent symptoms of parasarcoidosis include fatigue (70-80%), small-fiber neuropathy (30%), and cognitive impairment (35%). Included sequelae are “cognitive difficulties, such as impaired memory, slowed thinking, and diminished attention and concentration,” all in the setting of “no discernible evidence of nervous system inflammation" [11].

Even though parasarcoidosis affects over 50% of sarcoidosis patients, there is a lack of available literature on its management. One study has suggested that its pathogenesis is related to a systemic increase in tumor necrosis factor-α (TNF-α), a cytokine seen in inflammatory states that directly impacts neurotransmitter metabolism and synaptic transmission. This same study showed an improvement in cognition with anti-TNF-α medications, while patients who were treated with steroids +/- MTX did not show any improvement, further supporting the theorized mechanism of parasarcoidosis.

As seen in this study, sarcoidosis and lymphoma can present with similar systemic manifestations, with the ultimate diagnosis usually confirmed by biopsy. Although other studies have demonstrated a similar diagnostic dilemma, our patient's case was further complicated by his cognitive decline. Given a lack of active NS findings on MRI, a diagnosis of parasarcoidoiss was considered. Additional studies are needed to help elucidate both a diagnostic criterion for parasarcoidosis and measures for its optimal management.

## Conclusions

Sarcoidosis is a chronic granulomatous disease of unknown cause that presents with highly variable symptoms, especially when patients demonstrate extrapulmonary manifestations. The typical B symptoms of fatigue, weight loss, night sweats, and lymphadenopathy observed in sarcoidosis are often confused with lymphoma and other malignancies. These must be distinguished from lymphoma with histologic evidence of noncaseating granulomas. Other fungal, bacterial, and viral pathologies should also be excluded. While the JAMA Neurology guidelines published in 2018 improved the diagnostic schema for NS diagnosis, our case highlights the continued ambiguity in distinguishing NS from parasarcoidosis. Cognitive decline is an under-recognized but important symptom of sarcoidosis, especially given the expected improvement with appropriate therapeutics. Further investigation is warranted to help determine more specific testing methods for parasarcoidosis. Additionally, while TNF-α agents have been shown to be promising therapeutic agents for the sarcoidosis penumbra, more research is needed to properly determine the mechanism of parasarcoidosis in order to help guide future therapy.
